# Supplementary material for: The Temporal and Geographical Dynamics of Potato Virus Y Diversity in Russia
Source: Int J Mol Sci. 2023 Oct 2;24(19):14833. doi: 10.3390/ijms241914833 (PMC10573581; doi:10.3390/ijms241914833)
Supplement: Supplementary file 1 [file ijms-24-14833-s001.zip › ijms-2603006-supplementary.pdf]

International Journal of
*Molecular Sciences*

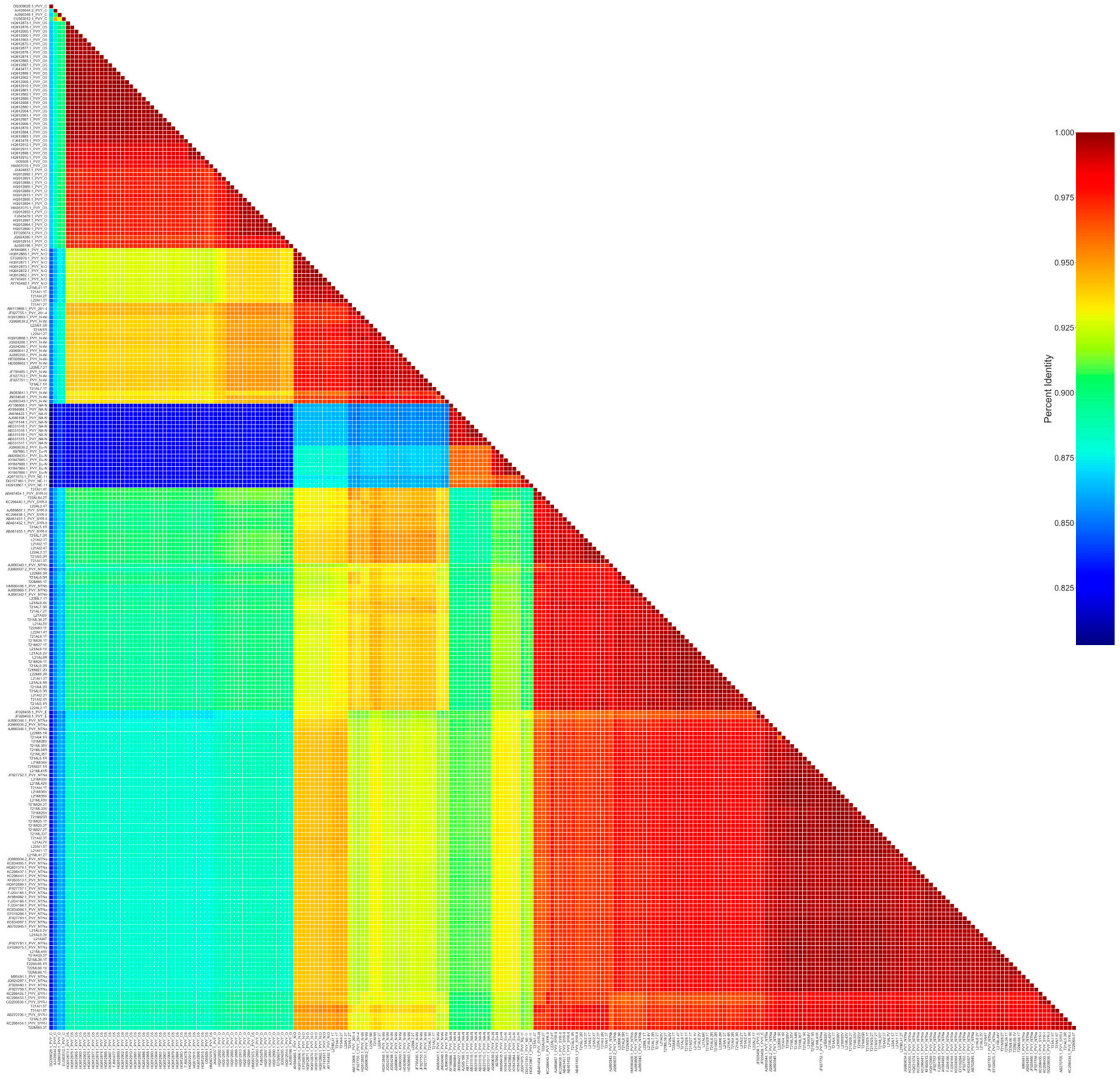


**Figure S1.** Color-coded pairwise identity matrix of the full-length PVY nucleotide sequences.

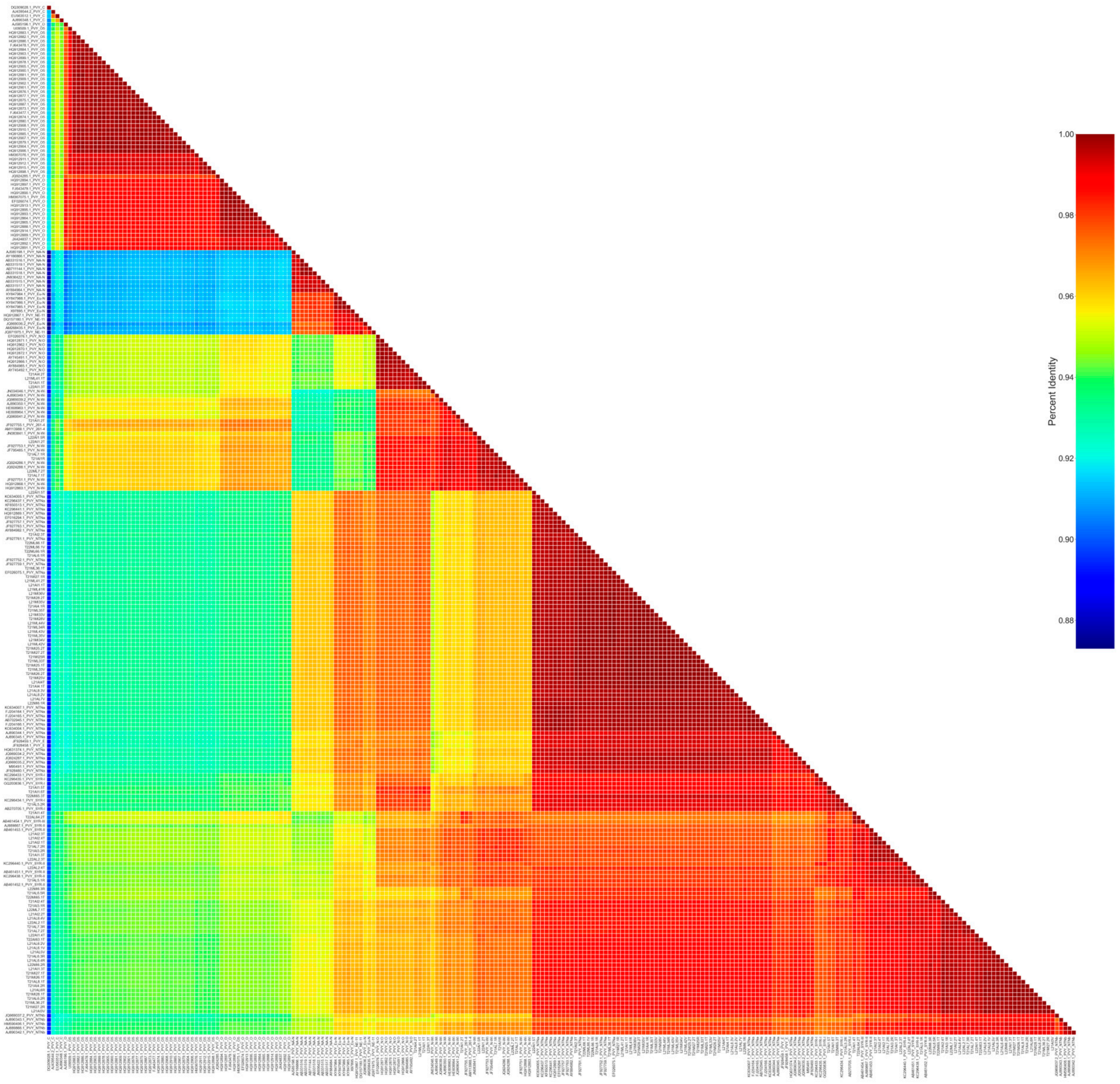


**Figure S2.** Color-coded pairwise identity matrix of the complete PVY polyprotein sequences.

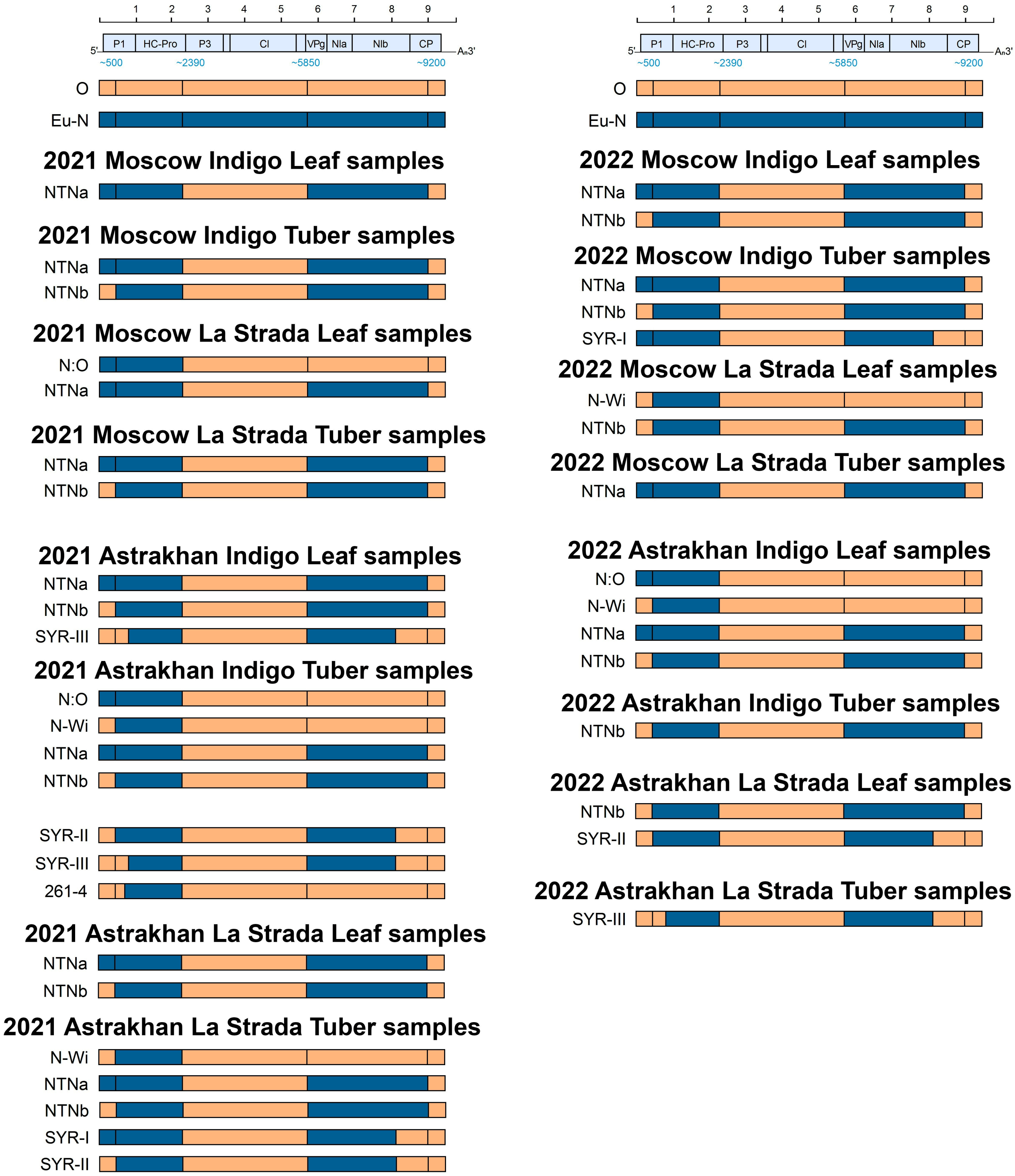


**Figure S3.** Schematic diagram of the known recombinant structures of potato virus Y (PVY) found in samples from the Moscow and Astrakhan regions, as well as five new recombinants described in this paper,
